# Graphene/PVDF Nanocomposite-Based Accelerometer for Detection of Low Vibrations

**DOI:** 10.3390/ma16041586

**Published:** 2023-02-14

**Authors:** Surendra Maharjan, Victor K. Samoei, Ahalapitiya H. Jayatissa

**Affiliations:** Nanotechnology and MEMS Laboratory, Department of Mechanical, Industrial, and Manufacturing Engineering (MIME), The University of Toledo, Toledo, OH 43606, USA

**Keywords:** flexible piezoresistive sensor, proof mass, low-frequency, accelerometer

## Abstract

A flexible piezoresistive sensor was developed as an accelerometer based on Graphene/PVDF nanocomposite to detect low-frequency and low amplitude vibration of industrial machines, which may be caused due to misalignment, looseness of fasteners, or eccentric rotation. The sensor was structured as a cantilever beam with the proof mass at the free end. The vibration caused the proof mass to accelerate up and down, which was converted into an electrical signal. The output was recorded as the change in resistance (response percentage) with respect to the acceleration. It was found that this accelerometer has a capability of detecting acceleration up to 8 g_pk-pk_ in the frequency range of 20 Hz to 80 Hz. The developed accelerometer has the potential to represent an alternative to the existing accelerometers due to its compactness, simplicity, and higher sensitivity for low frequency and low amplitude applications.

## 1. Introduction

Microelectromechanical system (MEMS)-based accelerometers have gained demand in different applications since piezoresistive-based MEMS accelerometers were first used in the automotive industry in 2000 for car suspension systems and airbags systems. Accelerometers measure the mechanical stimulation of a device in single or multiple axes in terms of acceleration, vibration, shock, and tilt and convert them into electrical signals. MEMS accelerometers are one of the highly efficient sensors [1] that lead to their expansion in a multitude of a branch of science. For instance, several applications of accelerometers are navigation systems of the aerospace and aviation industry, stabilization of unmanned aerial vehicles (UAVs), detection of industrial vibration of machinery, display adjustment of smart devices, bioengineering to detect subtle vibrations in rehabilitation [2], and activity tracking [3].

Based on the working mechanisms, MEMS accelerometers are categorized into piezoresistive, piezoelectric, capacitive, tunneling, optical, and thermal types. However, piezoresistive accelerometers hold simple circuit configuration, low power consumption, and fast frequency response that enhances their importance among others. The gauge factors of most piezoresistive metals and semiconductors used in the strain gauges are around 2. They are highly anisotropic for semiconductors due to the crystal orientation, dopant type, and carrier concentration [4,5]. Polymer/elastomer-based MEMS accelerometers have advantages such as ease of manufacturability, flexibility, simple configurations, optical transparency, and cost-effectiveness, which made them popular over metal and semiconductor accelerometers. The conductive materials such as graphene, carbon nanotubes, and metal particles are coupled with polymer/elastomer substrates such as PE, PMMA, PVA, PDMS, and ABS to make flexible sensor devices.

Detection of low-frequency vibrations is crucial in many applications. Vibrations in large rotating machinery, oil pipes [6], bridges, buildings, pillars [7], and ground motions caused by earthquakes, volcanic eruptions, explosions, landslides, tsunamis, and avalanches [8] hold a low frequency and low amplitude. Misalignment of components, mechanical looseness (nut, bolt, and fastener), and unbalance (about a central axis) are the main causes of vibrations in machines, which can cause lethal damage if not detected and maintained at the appropriate time. Accelerometers should have high sensitivity and reliability for such measurements. Nowadays, different research is being conducted to explore novel sensing materials, miniaturization techniques, sensitivity, reliability, and durability.

Table 1 depicts different types of accelerometers used for the detection of low frequency and low amplitude vibrations. Piezoelectric (PZT) accelerometers have a wide bandwidth and high sensitivity. Therefore, they are usually not applicable for low-frequency vibrations. Because of their high stability and low price, strain gauge accelerometers are widely used in industry. However, the accuracy is low to detect low-frequency vibrations. Fiber Bragg Grating (FBG) accelerometers can detect the vibration of low frequency but their resolution is limited. A MEMS accelerometer has a wide frequency response range and quite better sensitivity and acceleration (*g*) range than the previous types. In addition to the above technologies, Zheng et al. [9] developed a new maglev sensor that has a hybrid magnet levitation structure with the supporting components of electromagnets and permanent magnets. This accelerometer can measure an ultra-low frequency (ULF) vibration. A polysilicon-based piezoresistive MEMS accelerometer has been developed in recent years [10]. It has a capability to detect different frequencies from 75 Hz to 475 Hz as sinusoidal signals. Polymer composite materials made of Poly (Vinylidene-Trifluoroethylene) have been successfully used to develop an accelerometer, which has sensitivity in 20–50 Hz range [11]. However, we did not find any report about development of piezoresistive accelerometer based on Graphene/Polyvinylidene Fluoride (Gr/PVDF) composite.

In this study, a piezoresistive MEMS accelerometer with a novel material having high sensitivity, cost-effectiveness, and simple configuration, was developed for measuring low-frequency and low-amplitude vibrations. Gr/PVDF composite was used as conductive material and was coated on a polyethylene (PE) substrate to develop this flexible accelerometer [21]. The device was designed as a cantilever structure, which can vibrate and generate a change in resistance. This accelerometer can be calibrated for frequencies based on resistance change.

## 2. Sensing Mechanism

The piezoresistive accelerometer consists of a cantilever beam of substrate material coupled with sensing nanocomposite, which holds the proof mass at the free end and the other end supported by the fixed frame. This model has one degree of freedom and displace in the direction normal to the sensor surface. When the sensor is subjected to acceleration, a force equal to the product of mass and acceleration causes the proof mass to displace in the direction normal to the sensor surface [1]. According to Newton’s second law of motion, acceleration (*a*) is the function of displacement (*x*) of the proof mass and is given by [22],

(1) $$a=f\left(x\right).\;a=\frac{k}{m}x,$$

where *k* and *m* are the spring constant and mass, respectively. The resistivity of nanocomposite at the sensor legs will change, which in turn changes the resistance. The final output was taken as resistance change in this paper instead of voltage calculation to simplify circuit configuration. Thus, the acceleration applied to the sensor can be calibrated in terms of resistance change.

The change in resistance of the sensing material can be interpreted by the disconnection mechanism. The disconnection process between the adjacent nanoflakes is caused by three situations: contact area change, tunneling effect, and crack propagation. The change in the contact area between adjacent nanoflakes is dominant when the applied pressure or strain is small, and the electrons travel through the overlapped nanoflakes within the percolation conductive network. As the applied pressure increases, the adjacent nanoflakes pull apart and create a tunnel. However, electrons can pass through the tunnel due to very small separation. This is called the tunneling effect, and the separation space is called the tunneling distance. As the distance grows, so does the tunneling resistance. The distance at which no electron passes through by tunneling is called the cut-off tunneling distance. The tunneling resistance between two adjacent particles can be estimated by using Simmons’ theory [23].

(2) $$R_{tunnel}=\frac{h^{2}d}{Ae^{2}\sqrt{2m\lambda }}\;exp\left(\;\frac{4\pi d}{h}\sqrt{2m\lambda }\;\right)$$

where *A*, *e*, *h*, *d*, *m*, *λ* represent the cross-sectional area of the tunneling junction, single-electron charge, Plank’s constant, the distance between adjacent nanoflakes, the mass of an electron, and the height of energy barrier for insulators, respectively. The third one is crack propagation, which occurs when the applied pressure or strain is even higher. Initially crack initiates and later propagates along with time and pressure conditions. The separation of crack edges critically limits the electrical conduction. The sensors developed in this thesis are based on the piezoresistive mechanism.

## 3. Experimental Method

### 3.1. Materials Preparation

Graphite particles were mixed with acetonitrile as a solvent in the ratio of 1.0 g: 20 mL [24]. Large particles of graphite were broken down by rod stirring in the solution before transferring to the ultra-sonicator. It was sonicated 4 times, each for 10 min, keeping an interval of 10 min to prevent aggregation of graphite particles, which can be caused by temperature rise during sonication. The solution was kept at steady condition in a long measuring cylinder for a few hours (usually 12 h) to settle heavy particles at the bottom and 2.5 mL of suspended graphene layer was taken to the small beaker [25,26]. It was estimated from Raman spectroscopy and SEM images that 4–5 monolayer thick graphene is produced by this method. To prepare PVDF solution, 50.0 mg of PVDF powder (Alfa Aesar, 99.99%) was added acetonitrile and sonicated 2 times with the same procedure. Finally, graphene and PVDF solutions were mixed and sonicated for 20 min to achieve a homogeneous Gr/PVDF solution of the nanocomposite, which acts as the sensing element.

**Figure 1:**
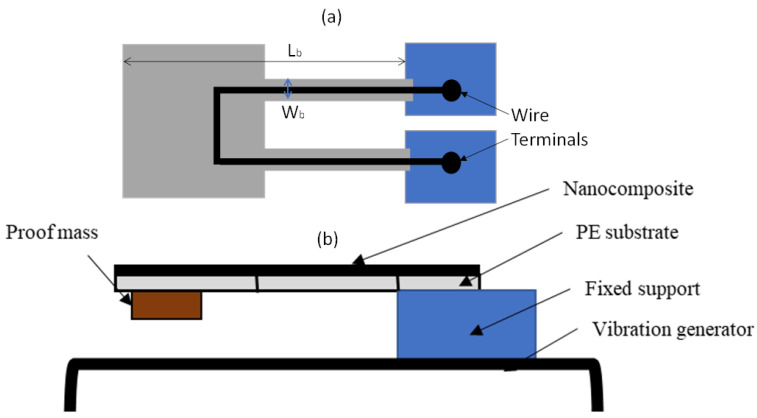
Accelerometer and experimental setup. (**a**) Top view and (**b**) side view of cantilever.

A thin and transparent flexible polyethylene sheet of thickness 0.1 mm was chosen as the substrate. The substrate was cut into 10 mm × 10 mm size to design a sensor as shown in Figure 1. The substrate was rubbed with sandpaper to better adhere the composite to the surface and then rinsed with isopropanol to wash away any contaminants before applying 20–22 µm thick graphene/PVDF nanocomposite using a doctor blade method.

### 3.2. Design and Fabrication of Accelerometer 

The sensor was designed as a cantilever structure (Figure 1) and one end of the sensor was attached to the proof mass while the other end was attached to the vibrator using an epoxy resin. The vibrator has a capability to produce different frequencies and amplitudes. Some space was maintained to freely vibrate the free end of the sensor along with the proof mass. The thin width legs of the sensor also support for ease of vibration. The vibration was only in the direction normal to the sensor surface. Two-wire terminals were attached to the sensor legs to record electrical signals using a Keithley Multimeter.

### 3.3. Resonance Frequency Calculation

The resonance frequency (*f*) of the system is given by Equation (3) [27],

(3) $$w^{2}{}_{n}=\frac{k}{m},\;f=\frac{1}{2\pi }\sqrt{\frac{k}{m}}.$$

The spring constant of the π-shaped structure follows the following equation [28].

(4) $$k=\frac{1}{2}Et_{s}{\left(\frac{W_{b}}{L_{b}}\right)}^{3},$$

where *E* is the Young’s modulus for PE substrate, *W_b_* is the width of the beam, *L_b_* is the length of the beam, and *t*_s_ is the thickness of the beam. The geometric parameters and accelerometer characteristics are shown in Table 2. Using Equations (2) and (3), spring constant and resonance frequency were found to be 56.7 N/m and 85 Hz, respectively.

## 4. Result and Discussion

### 4.1. Acceleration Response

**Figure 2:**
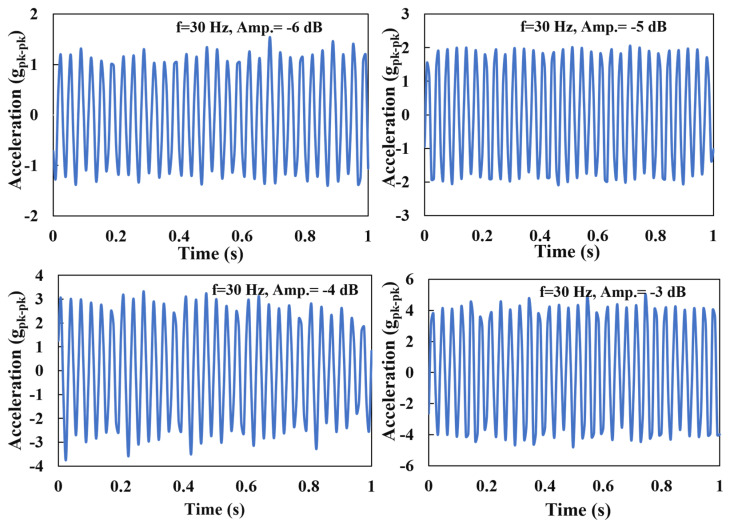
Results of acceleration measurement at 30 Hz and amplitudes ranging from −6 dB to −3 dB.

The device was placed in a vibrator as shown in Figure 1. One end of the device was fixed to the vibrator and the other end was kept free. The device was excited with a sinusoidal loading and the rectangular proof mass at the free end oscillates up and down, which is perpendicular to the sensor surface. The acceleration of the vibration was measured with respect to time. It was found that the sensor could measure the acceleration up to 8 g_pk-pk_ (peak to peak). For a representative case, a frequency of 30 Hz and amplitude from −6 dB to −3 dB was chosen for the excitation of the device. The results are depicted in Figure 2, which indicates that the device could convert a vibrational signal into acceleration.

**Figure 3:**
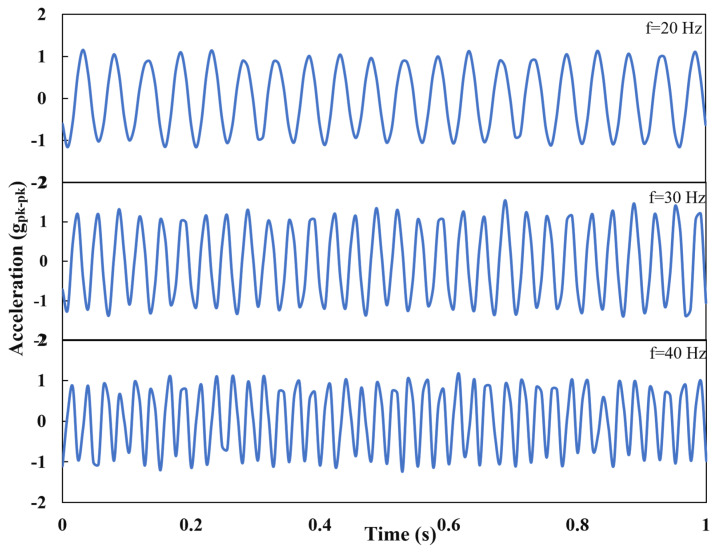
Time response of acceleration at three representative frequencies.

Controlling the acceleration of the vibration, the frequency was varied, and three representative cases were studied. Acceleration was kept at 2 g_pk-pk_ and frequency was set to 20 Hz, 30 Hz, and 40 Hz. Time responses of acceleration at three representative frequencies were recorded by the device as shown in Figure 3.

### 4.2. Resonance

**Figure 4:**
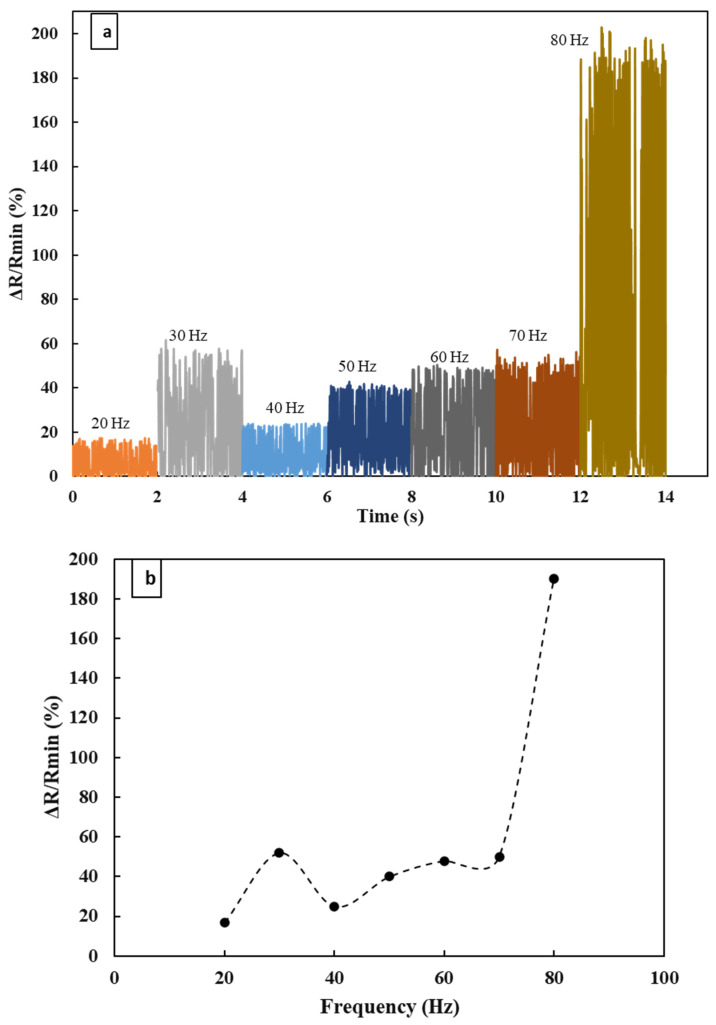
(**a**) Repeatability test. (**b**) Response measurement at different frequencies with a fixed amplitude of −5 dB.

The resonance frequency was calculated theoretically and was found to be 85 Hz. Usually, the MEMS accelerometer works below the resonance frequency. The lower limit of the vibration generator is 20 Hz, which is the lower possible limit of the device. The device was excited with a sine waveform signal with a variable frequency ranging from 20 to 80 Hz with an interval of 10 Hz. From 20 Hz to 60 Hz, data were collected for higher amplitude (−5 dB) whereas, for 70 Hz and 80 Hz, data were recorded at smaller amplitude (−9 dB). When the amplitude is higher at a higher frequency, vibration becomes so high that it damages the sensor. Figure 4a presents the repeatability curves in the experimental frequency range over a certain time frame. The higher response at 80 Hz was due to the proximity to the resonance. However, the data were displayed over 2 s for each frequency and each sinewave lies within the range. When the sensor was tested at 85 Hz, the vibration of the proof mass was so high that the sensor was damaged. Even after multiple testing, the sensor could not read the data, validating the calculated resonance frequency.

### 4.3. Sensitivity

**Figure 5:**
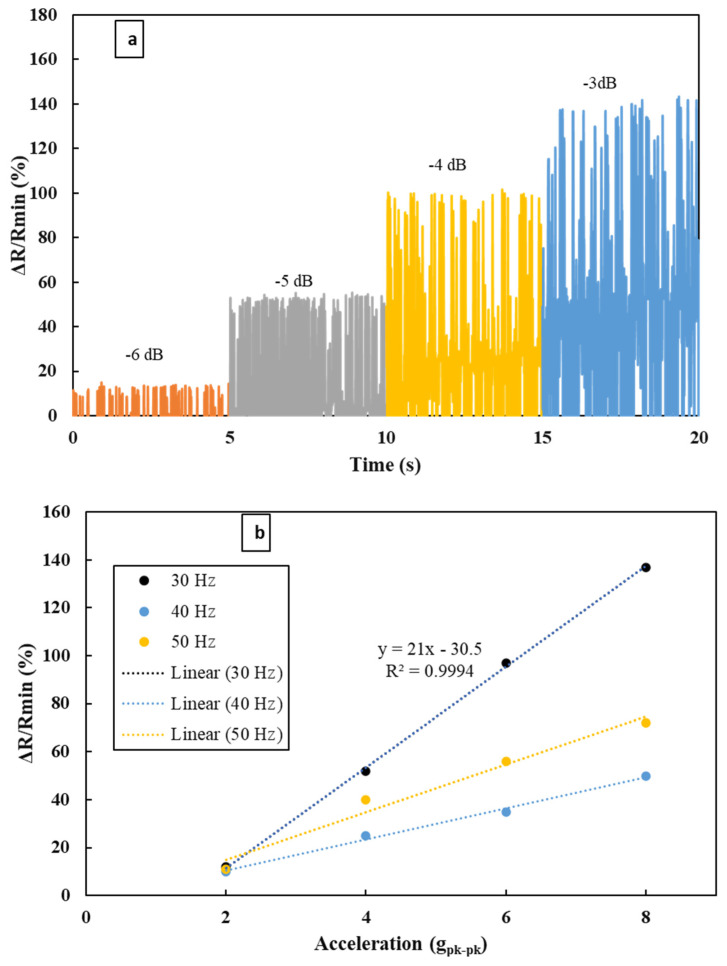
(**a**) Repeatability test at a representative frequency of 30 Hz for different amplitudes ranging from −6 dB to −3 dB. (**b**) Response measurement at a representative frequency of 30 Hz, 40 Hz, and 50 Hz for different g values.

The accelerometer was tested at a particular frequency varying the amplitude of the excitation. Figure 5a presents the repeatability of the curve lying within the range over a period and the data were displayed for 5 s for each amplitude. The highest sensitivity of the accelerometer was found to be 21% g^−1^ at 30 Hz with a Pearson squared correlation coefficient of R^2^ = 0.999, presented in Figure 5b. This means the response of the accelerometer can reach up to 21%, when the system is excited with 1 g_pk-pk_ at 30 Hz. The response was carried out in terms of resistance change. It was found that the correlation between acceleration and response (in terms of resistance change) is linear as the data were also recorded for 40 Hz and 50 Hz. The response in resistance change simplifies the circuit configuration.

The vibrations generated by machines can be dangerous if left unmarked as they can grow with time, leading to catastrophic damage to the system. The very common sensors that used to detect vibrations are piezoelectric accelerometers. These accelerometers are costly and based on piezoelectric material, which is very limited. This proposed piezoresistive sensor generates electrical resistance change instead of electrical voltage or current when there is a change in motion or acceleration. As observed in Figure 5b, the vibrational input of the vibrating machine in terms of acceleration (g_pk-pk_) and frequency can be correlated to the output in terms of change in resistance.

## 5. Conclusions

Accelerometers have been an indispensable part of most smart devices along with application in various fields. Nowadays, high-tech accelerometers are being used in the market with features including high sensitivity, light weight, durability, broad bandwidth, and cost-effective. As a novel material for sensing elements, this accelerometer possesses other features such as simple sensor configuration, light weight, and ease of fabrication. This accelerometer has the potential to detect a low-frequency vibration ranging from 20 Hz to 80 Hz for the acceleration up to 8 g_pk-pk_. The resonance frequency was found to be 85 Hz. This low-frequency vibration accelerometer can be implemented in machinery for recording vibrations caused by misalignment and unbalanced and mechanical looseness. This work is the first time investigating the application of the piezoresistive effect of graphene-based nanocomposite to develop an accelerometer covering a wide range of low frequencies. We believe that 2D materials are the best candidate for developing piezoresistive devices due to their stretchability and mechanical strengths. Further work is essential to realize the full potential of graphene/polymer composites in the development of piezoresistive MEMS accelerometers.

## Figures and Tables

**Table 1:** Summary of accelerometers for low vibration detection.

Principle	Sensitivity (V/g)	Range (g)	BW (Hz)	Ref.
PZT	9 mV/g	–	–	Tian et al. [12]
	15.6 mV/g	–	60–1.5 k	Nishshanka et al. [13]
	2.82	–	2–500	Tims et al. [14]
Strain	-	0–5	<100	Kamentse et al. [15]
FBG	0.135	0.1–2	80–800	Gao et al. [16]
	0.362	<0.5	1–10	Zhang et al. [17]
MEMS	2	±1	0–50	Swartz et al. [18]
	1	±2	0–50	Cho et al. [19]
	1.2	±3	0.2–1500	Sabato et al. [20]
Others	-	-	0.2–0.4	Zheng et al. [9]

**Table 2:** Geometric parameters and accelerometer characteristics.

Parameters	Symbols	Values
Length and width of the spring beam	*W*_b_ × *L*_b_	1.5 mm × 10 mm
Mass	*m*	0.2 g
Young’s modulus	*E*	1.08 × 10^9^ Pa
Thickness of beam	*t*_s_	105 μm

## Data Availability

Not applicable.
